# The COVID-19 pandemic as experienced by the spouses of home-dwelling people with dementia – a qualitative study

**DOI:** 10.1186/s12877-021-02551-w

**Published:** 2021-10-20

**Authors:** Anne Marie Mork Rokstad, Janne Røsvik, Marit Fossberg, Siren Eriksen

**Affiliations:** 1 Norwegian National Advisory Unit on Ageing and Health, Vestfold Hospital Trust, Tønsberg, Norway; 2 Molde University college, Faculty of Health Sciences and Social Care, Molde, Norway; 3 Department of Geriatric Medicine, Oslo University Hospital, Oslo, Norway; 4 Lovisenberg Diaconal University College, Oslo, Norway

**Keywords:** COVID-19, Dementia, Informal caregivers, Spouses, Social isolation, Well-being, Health and social care services

## Abstract

**Background:**

Worldwide, restrictive measures have been taken to manage the spread of the COVID-19 pandemic. Social distancing and self-isolation have considerably affected the lives of people with dementia and their informal caregivers. The purpose of the study was to explore the consequences of the COVID-19 pandemic as experienced by the spouses of home-dwelling people with dementia in Norway.

**Methods:**

The study had a qualitative descriptive design using individual telephone interviews for data collection. A total sample of 17 spouses of people with dementia were included, 14 women and three men ages 52 to 82 years. A qualitative content analysis following six steps inspired by Graneheim and Lundman was used to identify the categories presented.

**Results:**

The participants emphasized four main perspectives: 1) Radical changes in available services, 2) Restrictions changed everyday life, 3) Impacts on health and well-being, and 4) Actions that made life easier. The participants also described how positive activities and easily accessible services helped them in this situation.

**Conclusions:**

The governmental restrictions of the COVID-19 pandemic resulted in radical changes in available services with severe consequences for the lives and well-being of home-dwelling people with dementia and their spouses. Examples of coping strategies and possible psychosocial interventions compatible with virus precautions were identified. The potential of such interventions should be further explored to meet the needs of vulnerable groups in situations like a pandemic.

## Background

In Norway, due to the COVID-19 pandemic outbreak, the government closed most activities in the society starting on 12 March 2020. Citizens were advised to remain at home as much as possible. As reported by Alzheimer Europe (2020), the restrictive measures implemented to attempt to limit the spread of the COVID-19 virus outbreak affected people with dementia and their caregivers as they were asked to isolate themselves [1]. In following these directives, they were cut off from their regular support system and had to change their routines. Social distancing and self-isolation constitute a challenging situation for most people, but limited social stimulation and interaction can be a particular threat to the well-being of people with dementia and their informal caregivers [1].

The dementia condition not only affects the person with dementia but also influences the person’s family and friends, particularly a spouse [2]. Dementia caregiving has been associated with negative effects on the physical, psychological, emotional and functional health of caregivers [3–5] and early nursing home placement for people with dementia [6]. According to the literature review of caregiver burden among dementia patient caregivers by Etters et al. (2008) several factors have impacts on the caregiving experience, including sex, relationship to the patient, culture, and personal characteristics [6]. Evidence suggests that individually tailored, multicomponent interventions including a diversity of services will decrease the care burden, improve quality of life, and enable caregivers to provide at-homecare to the person with dementia for longer periods before institutionalization becomes necessary [6]. Daycare services for the person with dementia create a break from caregiving tasks and reduce feelings of burden, worry and depression among next of kin [7]. In addition, such services may have a positive influence on the relationship between the next of kin and the person with dementia, leading to improved cooperation and higher quality of time spent together [7].

The period of self-isolation during the COVID-19 pandemic may have contributed to feelings of loneliness, behavioral changes and acute events of agitation or delirium in people with dementia [8]. Studies exploring the impact of the restrictions during the initial period of the pandemic reveal possible worsening of cognition, function and neuropsychiatric symptoms in this group [9]. Moreover, the duration of the isolation period seems to correlate with the severity of neuropsychiatric symptoms [10], and increased caregiver stress is also described [9, 10]. As most people with dementia are dependent on a combination of formal and informal care, the impact of the pandemic can influence their everyday lives in various ways. For some, in-home support and services have become unavailable due to increased demands and reduced capacity in the service due to health care personnel who are in quarantine, in recommended isolation or on sick leave. The pandemic may result in some family caregivers becoming ill, being unavailable or needing to isolate. Additionally, family caregivers risk developing anxiety, depression, or other mental health concerns. The situation could potentially lead to caregiver exhaustion and burnout [11].

Knowledge about the experiences of caregivers and people with dementia during the COVID-19 pandemic is scarce. One previous qualitative study by Giebel et al. (2020) investigated the impact of the closure of social support services on the target groups [12]. The study found that the participants experienced a loss of control, became increasingly uncertain, and revealed that few care services had adapted to the situation by providing remote support [12]. There is a need for more knowledge of the experience of COVID-19 in order to understand, learn and prevent possible serious consequences for this group in similar situations with possible endemics or pandemics in the future.

## Method

### Aim

The aim of this study was to explore the consequences of the COVID-19 pandemic as experienced by the spouses of home-dwelling people with dementia in Norway. The following research questions were formulated: 1) What consequences did the COVID-19 restrictions have for the health care and social care services offered to home-dwelling people with dementia and their spouses? and 2) What are the experiences of spouses of people with dementia related to the recommendations of social isolation and other virus-related restrictions?

### Design

The study had a qualitative, descriptive design. Individual interviews were considered an appropriate method to explore the experiences of spouses in order to obtain in-depth knowledge of their individual situation during the first 9 to 11 months of COVID-19 restrictions in Norway lasting from March 2020 to February 2021.

### Setting

In Norway, homecare services are provided as public statutory services. They are free of charge and offered 24 h a day, 7 days a week. Each municipality is financially responsible for providing primary health care services, including daycare, to their inhabitants. The provision of daycare has been one of the main priorities of the Norwegian government’s dementia-care strategy [13, 14] and starting in January 2020, all Norwegian municipalities were obliged by law to offer daycare to people with dementia. Daycare is offered mainly during the daytime hours on weekdays [15]. The Norwegian national guidelines for dementia strongly recommend that municipalities establish multidisciplinary teams with members who are skilled in dementia care, often called dementia coordinators [16]. They act as contact persons or case managers and provide post-diagnostic support to people with dementia and their next of kin [17, 18]. The main strategy to limit the severity of the COVID-19 pandemic by reducing contact between people lead to the lock down of major parts of the Norwegian society from March 2020. Public services like daycare services, group activities and cultural arrangements were closed and nursing home residents were not allowed to have visits [19]. There have been some variations in the level of restrictions, when nursing home visits have been permitted to some extent, but the strong recommendations for social distancing have been permanent during the last 1,5 year [20]. Norway has had low morbidity and mortality rates during the pandemic compared with other countries [21].

### Sample and recruitment

The participants for the present study were recruited by local gatekeepers, such as dementia coordinators and daycare service leaders, in the network of the Norwegian National Advisory Unit on Ageing and Health. The gatekeepers distributed information by mail about the study to spouses and partners of people with dementia on their contact list, with an invitation to participate in the study. Contact information for the research group was included, making it possible to sign up for participation. The criterion for inclusion was to be a spouse or partner of a home-dwelling person with dementia who was receiving health care and social care services. To ensure representation from a variety of communities, participants living in both urban and rural areas and in small, medium, and large municipalities were included. Three out of four health regions in Norway were represented. The sample comprised 17 spouses and partners (hereafter referred to as spouses), All participants gave their written informed consent.

### The telephone interviews

**Fig. 1:**
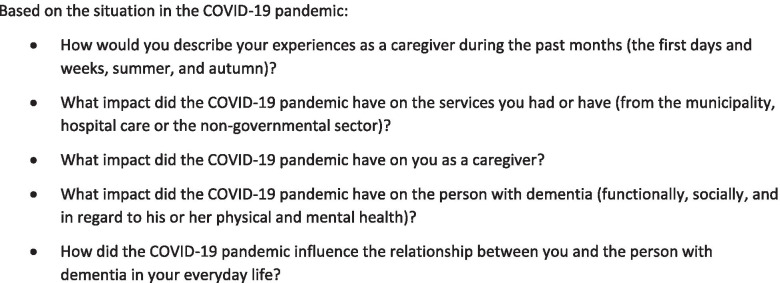
Interview guide

The interviews were conducted in line with a phenomenological-hermeneutic approach, and the participants were encouraged to share their experiences and describe how they felt during the months since the outbreak of the COVID-19 virus. The interviews were conducted by the co-author MF from December 2020 to February 2021. All interviews took place via telephone, were audio-recorded and transcribed verbatim. The participants were invited to express their experiences in open dialog with the interviewer based on a thematic interview guide (see Fig. 1). As the interviews were made in Norwegian language, the quotations used in this paper have been translated into English.

### Analysis

The transcribed interviews were analyzed according to qualitative content analysis inspired by Graneheim and Lundman [22]. Qualitative content analysis can be conducted on different levels of abstraction, and the content of a text can either be manifest describing the visible, obvious components, or latent involving an interpretation of the underlying meaning of the text [22]. The materials collected in this study were analyzed on a manifest level and presents what is directly expressed in the text, providing a description of its visible and tangible components [22]. The analysis was conducted in six steps: 1) All transcripts were read to provide a sense of the whole and themes that emerged from the interviews and were considered to contribute to the research questions were identified.; 2) meaning units in the text were identified; 3) the identified meaning units were condensed into descriptions close to the text and with the intention of maintaining the subjective perspective of each participant; 4) the meaning units were extracted and labelled with codes; 5) the codes were compared based on similarities and differences and grouped into subcategories; and finally, 6) the subcategories were grouped and abstracted as categories [22]. The first author (AMMR) performed the initial analysis following steps one through four. All authors took part in steps five and six.

## Results

The results are based on telephone interviews with 17 spouses, 14 (82%) women and three (12%) men. Their age ranged from 52 to 82 years, with a median age of 68.

The experiences of the participants emphasized four perspectives identified as categories; 1) radical changes in available services; 2) restrictions changed everyday life; 3) impacts on health and well-being; and 4) actions that made life easier (see Table 1).

**Table 1:** Categories and subcategories identified from the analysis

Categories	Radical changes in available services	Restrictions changed everyday life	Impacts on health and well-being	Actions that made life easier
Subcategories	The only one to support the person with dementia 24/7	Changed routines	The person with dementia changed	Personal initiatives
	Need for more services	A relationship in solitude	I came to the limit of what I could endure	Easily accessible services

### Radical changes in available services

The suspension of activities throughout the society in March 2020 resulted in radical changes to the services offered to home-dwelling people with dementia and, in turn, led to major changes for their caregivers.

#### The only one to support the person with dementia 24/7

In the first 3 months of the COVID-19 pandemic, the participants experienced being left alone with the responsibility for the person with dementia. Most of the health and social care services offered to the person with dementia disappeared or were significantly reduced from one day to the next. Most daycare centres and activity groups were closed; respite stays in residential care were interrupted, cancelled, or postponed; and visits from dementia coordinators, personal assistants and in-home physiotherapists were terminated. Additionally, support groups for informal caregivers came to a halt, and homecare nursing services were reduced. Furthermore, some participants refused to receive visits from health care professionals in their own homes based on their fear of infection. These changes resulted in extensive impacts on the lives of both persons with dementia who lost their services and the participants: ‘Everything was closed, so suddenly I was the only person to support my husband with dementia 24 hours a day, seven days a week’ (woman, age 64). Most participants experienced the shutdown of daycare services as losing their only break from their caregiving responsibilities. Some expressed a feeling of panic about getting sick as well as about being left alone with the full responsibility for providing care:

The first period was very frightening, there was a disease we knew nothing about and when the homecare nurses stopped coming in March, I became a mother, wife, nurse and support coordinator around the clock. Then, the daycare centre was closed, and I started crying. I didn’t have a minute free time. It was simply a month in hell. (woman, age 57)

In addition, the participants described the continuing deprivation experienced by the person with dementia who was now unable to attend daycare: ‘Every morning he asked, “Am I going anywhere today? And I had to say, “I am sorry, but the daycare is closed”. He really missed going there’ (woman, age 64).

After the first period of shutdown in the society, lasting three to four months, the participants experienced that health and social care services again became available for the person with dementia. Some regained their previous services, and others received alternative and more-flexible services like individual support from dementia coordinators: ‘We got a coordinator who visited us at home. My wife was not very enthusiastic about this to start with, but they developed a good relationship, and she (the coordinator) has been a great support’ (man, age 67).

#### A need for more services

After living with restrictions for several months, many of the spouses experienced a greater need for services than before the pandemic. Some spouses reported that they needed more services in order to be able to continue to take care of the person with dementia at home: ‘As a result of the burden of care I had during the first months of lockdown, we had an emergency meeting and my husband got regular respite stays in residential care’ (woman, age 64). Others had to ask for more days in daycare services or even admission to long-term nursing homecare as the person with dementia had become more dependent, with more-severe cognitive and functional impairments.

### Restrictions changed everyday life

Due to the pandemic’s restrictions, daily routines had to be altered, and possibilities to meet other people became limited.

#### Changed routines

In addition to the sudden absence of health and social care services, the COVID-19 restrictions and recommendations had significant impacts on the everyday lives of the participants and the persons with dementia. Common everyday tasks like going out to buy groceries were a challenge as it was difficult for the person with dementia to understand why he or she had to wear a mask, maintain a certain distance from other people and not touch things in the shop. Activities for leisure and critically needed cognitive and physical stimulation became limited, which was experienced as difficult: ‘We had to stop meeting in the choir …. That was a catastrophe for her’ (man, age 74). The closure of cultural activities and restaurants and the reduced opportunities for meeting friends and family isolated the couples at home:

We were locked up in the house, in our apartment 24 hours a day …. He understood so little of what was going on, and after a while, he only saw his own needs …. He didn’t see me at all. The total absence of impulses from the normal world brought him into the “Alzheimer world” …. Looking back on that period … it was a big black hole. (woman, age 64)

#### A relationship in solitude

The social restrictions forced the couples to be close together with limited stimuli from the outside, ‘normal’ world. This influenced the relationship between the participant and the person with dementia in different ways. One of the wives talked about a closer relationship with her husband with dementia: ‘He was more dependent on me; I felt sorrier for him, and the attachment became stronger between us, even though I was frustrated and stressed from time to time’ (woman, age 57). However, most participants experienced a more complicated relationship to the person with dementia, explaining how the burden of care had increased and how they struggled to keep the relationship going. The actions the participants needed to take in order to manage the formal restrictions influenced their communication negatively. Some participants felt they were nagging, and some persons with dementia responded with anger. Other participants talked about how the stressful situation made them angry and how their frustration accumulated until they lost their patience and raised their voices. One of them related her husband saying, ‘Can’t you stop using that angry voice?’ (woman, age 82).

In addition to reduced formal services, because of the restrictions the couples lost the much-needed regular support they had received from family and friends. The longing for being physically near their family members was mentioned by most participants. Limited possibilities for contact with their children and grandchildren increased the pandemic’s negative effects on the relationship. Due to the risk of infection, family members were afraid to visit the couple; hence, contact had mainly been via phone or digital social platforms. This could be difficult, especially for some persons with dementia: ‘We had digital contact with our children and grandchildren, but he found it difficult to know if they were here or not (while talking via FaceTime and Teams)’ (woman, age 66).

Several participants emphasized the lost opportunities to engage with friends. One husband described that his wife’s friends had disappeared. A wife said, ‘We have lost all our social network during these months because they (the friends) are in the risk group or have spouses in the risk group, so they don’t initiate contact anymore’ (woman, age 68).

### The impacts on health and well-being

The pandemic and the formal restrictions it required to prevent infection had impacts on the health and well-being of both the persons with dementia and their caregivers, as experienced by the participants.

#### The person with dementia changed

Some participants observed changes in the behaviour and cognitive and functional abilities of the person with dementia. As the restrictions on activities were difficult for the person with dementia to understand and comply with, several situations became stressful challenges for the participants in various ways. Some participants experienced that the person with dementia became extensively focused on certain activities like cleaning and other household tasks, while other participants experienced the opposite where the person with dementia became apathetic and passive. One husband said, ‘She just sat on the sofa all day long’ (man, age 60). Another stated, ‘She was more withdrawn, just sitting there wondering if anything was going to happen, mostly sleeping actually’ (man, age 67).

The limited possibility for meaningful activities and mental stimulation was a main challenge to maintaining cognitive and practical functioning: ‘From March to October, his functional level has decreased extremely. I am convinced that if it wasn’t for the COVID-19 pandemic, he could have sustained his daily routines and attended daycare, and his functional level would have been more stable’ (woman, age 57). Some participants were more uncertain about how the pandemic had influenced the person with dementia, especially when the cognitive impairment was more severe: ‘In fact, I don’t know what consequences the situation has had on my husband. He lives in his own world, so to speak. He doesn’t express anything, so I simply don’t know’ (woman, age 68). Additionally, physical activities and training were limited: ‘She enjoys going to the gym, but she has not had the same possibilities to go there as it has been closed for long periods of time. So, this last year, especially the last months, her disease has developed negatively.... I see more confusion and difficulties in orientation’ (man, age 60).

#### I came to the limit of what I could endure

Based on the loss of services and support, the changes in everyday routines and the increased responsibility and burden of care had considerable impacts on the health and well-being of the participants. They expressed becoming exhausted, burned out or physically ill. They described suffering from various depressive and anxiety symptoms. Some had a persistent feeling of stress, some developed insomnia, and some described emotional instability with irritability, impatience and a tendency to burst into tears easily. A wife said, ‘I am emotional and crying for every little thing that I never have cried for previously’ (woman, age 66).

### Actions that made life easier

Indisputably, the COVID-19 pandemic had significant negative impacts on the lives of the participants and the persons with dementia. However, several positive examples of actions that helped them to cope with everyday challenges emerged from the interviews.

#### Personal initiatives

Many participants described the importance of getting outdoors, going for a hike, walking the dog and seeking out nature. Some took advantage of the reduced risk for infection when meeting others outdoors and got together with close friends outside. Physical exercise was highlighted as important as coping strategies both for the persons with dementia and the participants.

#### Easily accessible services

In addition to the personal initiatives and actions to enhance well-being in the challenging situation of the COVID-19 pandemic, several examples of simple but important actions initiated by health and social care services were highly appreciated by the participants. For instance organized walking groups for people with dementia as an alternative to daycare were mentioned as a positive service: ‘When the walking group was offered, it was like coming out of prison for me’ (woman, age 68). The continuation of group meetings for family caregivers organized as a professional service, by voluntary organizations or by personal initiatives by friends or family were mentioned as important sources of support. Regular calls from professional staff made an immense difference for one of the participants. ‘Because the situation was so unclear and seemed endless, the weekly call from the daycare centre asking how she was coping helped her to persevere in this situation as this contact represented hope and encouragement in everyday life’ (man 67).

## Discussion

Together, the four categories: 1) radical changes in available services; 2) restrictions changed everyday life; 3) impact on health and well-being; and 4) actions that made life easier, describe how the pandemic affected the lives of the spouses of home-dwelling people with dementia. Even though there have been some variations in the level of restrictions in the Norwegian society since March 2020, the strong recommendations for social distancing, were present for more than a year. It can be challenging for next of kin and people with dementia to accept formal care as it is perceived as a threat to the individual’s independence and, thus, will be avoided as long as possible [23]. When they finally accept formal care, it is highly needed. Our findings illustrate how far the spouses go to take care of their loved ones and how losing services like daycare, respite and visits from dementia coordinators influences their situation dramatically. Studies have also found that support and service arranged by the municipality for people with dementia living at home is essential for the well-being of the spouse. Daycare services have, for instance, been shown to provide a feeling of safety and relief, to increase motivation and to decrease the burden of care for next of kin [24, 25]. Our participants outlined the benefits of positive actions to increase well-being and measures of support that helped them to keep going during the pandemic.

Previous research has revealed that burden of care is associated with being female, living together with the person with dementia, poor physical and mental health, limited support and higher numbers of hours spent on caregiving [3, 6, 26–29]. Hoffart et al. (2020) investigated loneliness in relation to the social distancing strategy of the pandemic [30]. In adult populations, loneliness was associated with both depression and anxiety. Among the stated risk factors, more rumination and worry in general were associated with greater feelings of loneliness [30]. These findings could be relevant for the spouses, being in increased risk of depression and anxiety, as they experienced extended worries and stress in their everyday-life in isolation together with the person with dementia. Giebel et al. (2020) have investigated people with dementia and their carers during the COVID-19 pandemic [12]. They emphasized the need to provide specific practical and psychological help during the pandemic to support caregivers’ well-being, which is severely affected by public health restrictions [12]. The participants in the current study expressed how the extraordinary pandemic situation was leading to psychological instability such as depressive and/or anxiety symptoms. Findings from a survey in by Altieri and Santangelo (2021) of 84 caregivers of people with dementia looked at depressive symptomatology and anxiety before and during the COVID-19 lockdown [31]. The survey revealed increased levels of depressive symptoms among the participants. Based on the findings, Altieri and Santangelo recommend that all caregivers of people with dementia, even those with high resilience levels, be provided with psychological interventions to reduce levels of depression, anxiety and caregiver burden [31]. The findings of caregiver burden in our study, revealing possible increased risk for depression and anxiety among spouses, seem to support this recommendation. Alternative interventions to support spouses of home-dwelling people with dementia could be regular contact and support from the general practitioner, a psychologist, a dementia coordinator or organized support groups.

The COVID-19 pandemic has been experienced differently across individuals, and older adults’ different life experiences have led to a variety of coping strategies. The survey by Whitehead and Torossian (2021), which included 825 adults age 60 and older, revealed that stress about concern for others and the experiences of how to manage and plan for an unknown and unforeseen future were significantly associated with poorer psychological well-being [32]. This supports the findings in our study revealing the stressful situation of being alone with the full responsibility for the care and well-being of a person with dementia. The feeling of deprivation and constraints in daily-life activities from COVID-19 restrictions seem general and similarly felt across nations as illustrated in the study by Gonçalves et al. (2021) reporting findings from 24 qualitative interviews with older adults in four different countries [30]. The findings revealed a homogeneity in the statements, showing that the pandemic affected participants similarly, regardless of the social and/or cultural context and the country in which they live [33].

It is argued that people with dementia need special attention in the demanding times of the COVID-19 pandemic as they may find it more difficult to comply with protective measures needed to limit the spread of the virus [34]. This statement was fully supported by the findings in our study. Although the participants supported the protective measures initiated by the authorities, they still had to cope with the consequences of the restrictions almost on their own, especially in the first months of the pandemic. The well-being and rights of persons with dementia have been at particular risk during the pandemic, and this applies to their caregivers as well [1]. In Norway, the local authorities’ obligation to offer day activities to people with dementia was more or less set aside during the state of emergency that began in March 2020, illustrating the risk of their human rights and basic needs being downgraded.

Couples where one of the partners suffers from dementia often have a strong sense of commitment to each other and make great effort to maintain a sense of togetherness [35]. But dementia could both preserve and challenge the value of ‘us’ [36] and there are reasons to believe that the couplehood also is vulnerable for outer changes. The participants in our study described that being isolated together at home challenged the relationship they have with their spouse with dementia. Being a caregiver for a person with dementia has been associated with different kinds of negative effects in “normal times” [3–5]. It is not hard to imagine that the forced “being on your own” could make it difficult to maintain the sense of togetherness as there has been no pause and no respite for the spouse. In addition, the cognitive, behavioural and physical function deteriorated during the pandemic for some of the persons with dementia.

The possibility to get outdoors was one of the positive experiences stated by the participants. This is in line with the findings of Whitehead and Torossian showing that having contact with nature was associated with greater positive psychological well-being during the pandemic [32]. Furthermore, the benefits of engaging in outdoor activities correspond with the findings of Beerens et al. (2018), who reported that activities outdoors, even if only observing others, contributed to well-being and improved mood in people with dementia [37]. A systematic review including both quantitative and qualitative studies reported that participating in physical activity and spending time outdoors had a positive influence on mood and well-being and the ability of people with dementia to maintain their sense of self [38].

### Limitations

The use of telephone interviews to collect the empirical data could influence participants’ confidence and willingness to share their personal experience. The development of a trusting relationships with participants may be more difficult over the telephone. However, telephone interviews appear to be an appropriate interview method as they may be more effective when the need for anonymity is high [39]. This study includes 17 participants sharing their personal experiences of their situation during a period of COVID-19 restrictions and refers to a Norwegian context. However, the purpose of a qualitative study is not to generalize but rather to understand the phenomenon more in depth. Compared with previous research and as a possible study to include in a future meta synthesis of qualitative studies on this topic, the study could contribute to extended knowledge on the impact of the COVID-19 pandemic on people with dementia and their spouses.

## Conclusion

This study shows that the restrictions of the COVID-19 pandemic resulted in radical changes in available services that had severe consequences for the everyday life and well-being of home- dwelling people with dementia and their spouses. From 1 day to another, the spouses were left alone with the responsibility for caring for loved ones with dementia. However, the findings also show that it is possible to establish coping strategies and psychosocial interventions that are compatible with virus precautions and may alleviate their impact. The findings reveal the extended need for practical and psychological support from health professionals to people with dementia and their spouses during a period of social restrictions in the society. Regular contact could be made from general practitioners and dementia coordinators via phone calls or digital media. Meeting outdoors for a walk or a picnic could be an alternative to traditional daycare. More research is needed on services and support systems that can be maintained for vulnerable groups in situations like a pandemic.

## Data Availability

The datasets used and/or analyzed in relation to the current study are available from the corresponding author upon reasonable request.
